# Plasma RANKL levels are not associated with breast cancer risk in *BRCA1* and *BRCA2* mutation carriers

**DOI:** 10.18632/oncotarget.26810

**Published:** 2019-03-29

**Authors:** Tasnim Zaman, Ping Sun, Steven A. Narod, Leonardo Salmena, Joanne Kotsopoulos

**Affiliations:** ^1^ Department of Pharmacology and Toxicology, University of Toronto, ON, M5S 1A8, Canada; ^2^ Women’s College Research Institute, Women’s College Hospital, Toronto, ON, M5S 1B2, Canada; ^3^ Dalla Lana School of Public Health, University of Toronto, ON, M5T 3M7, Canada; ^4^ Princess Margaret Cancer Centre, University Health Network, Toronto, ON, M5G 2M9, Canada

**Keywords:** receptor activator of nuclear factor κB (RANKL), breast cancer, biomarker, BRCA

## Abstract

**Background:**

Aberrant progesterone/receptor activator of nuclear factor κβ (RANK) signaling has been implicated in *BRCA1* breast cancer development. Furthermore, lower circulating RANKL has been reported among women with a *BRCA* mutation compared to non-carriers; however, there have been no reports of plasma RANKL levels and subsequent breast cancer risk. We prospectively evaluated the relationship between plasma RANKL and breast cancer risk among women with a *BRCA1* or *BRCA2* mutation.

**Methods:**

An enzyme-linked immunosorbent assay was used to quantify plasma RANKL levels in 184 *BRCA* mutation carriers. Women were stratified into high vs. low RANKL based on the median levels of the cohort (5.24 pg/ml). Kaplan-Meier survival analysis was used to estimate the cumulative incidence of breast cancer by baseline plasma RANKL and cox proportional hazards models were used to estimate the adjusted hazard ratios (HRs) and 95% confidence intervals (CI) for the association between plasma RANKL and risk.

**Results:**

Over a mean follow-up of 6.3 years (0.02-19.24), 15 incident breast cancers were identified. The eight-year cumulative incidence was 10% in the low RANKL group and 12% in the high RANKL group (*P*-log-rank = 0.85). There was no significant association between plasma RANKL levels and breast cancer risk (multivariate HR high vs. low = 1.06; 95%CI 0.34-3.28; *P-*trend = 0.86).

**Conclusions:**

These findings suggest that circulating RANKL levels are not associated with breast cancer among *BRCA* mutation carriers. Pending validation in a larger sample, these findings suggest that RANKL is likely not a biomarker of breast cancer risk among *BRCA* mutation carriers.

## INTRODUCTION

The receptor activator of nuclear factor κB (RANK), its cytokine ligand (RANKL) and the soluble receptor osteoprotegerin (OPG) are proteins of the tumor necrosis factor (TNF) and TNF receptor superfamily [1, 2]. They are important cell signaling factors which have many downstream effects on many tissues. Emerging evidence strongly implicates a role of the RANK signaling pathway in both normal mammary gland development and carcinogenesis [3–5]. Namely, progesterone mediated activation of the RANK signaling pathway promotes proliferation, differentiation, and migration of mammary epithelial cells [4, 6] as well as expansion and survival of mammary stem cells [7]. In contrast, OPG is the endogenous decoy receptor for RANKL that antagonizes RANK signaling [2]. Importantly, two key preclinical studies recently demonstrated that inhibition of RANKL significantly suppressed *Brca1*-mammary carcinogenesis suggesting that this may be a novel target for prevention in women at a high risk of developing breast cancer due to an inherited *BRCA1* mutation [8, 9]. Furthermore, Widschwendter *et al.*, reported significantly lower circulating levels of osteoprotegerin (OPG) and RANKL, as well as higher circulating progesterone levels, among premenopausal *BRCA* mutation carriers compared to non-carrier controls indicating possible dysregulation of sex hormones and RANK signaling in this population [10].

We recently published a significant inverse relationship between plasma OPG levels and breast cancer risk in a cohort of 206 *BRCA* mutation carriers [11]. Our findings suggest that circulating OPG levels may serve as a biomarker to identify those at the highest risk of developing breast cancer risk and who may benefit most from chemoprevention with RANKL blockade [11]. Among women in the general population, there is some evidence to suggest that high serum RANKL levels are associated with an increased risk of ER-positive, but not ER-negative, disease [12]. To our knowledge, there have been no evaluations of circulating RANKL levels and breast cancer risk among women with a *BRCA1* or *BRCA2* mutation.

Women with an inherited *BRCA* mutation face high lifetime risks of breast and ovarian cancer [13]. Prophylactic surgery remains the gold standard to prevent disease and improve outcomes [14, 15]. The elucidation of a biomarker that can identify those at the highest risk of developing disease while providing insight into the underlying mechanism(s) of cancer development are necessary. Given that aberrantly lower levels of circulating RANKL have previously been described among women with a *BRCA* mutation, along with the important role of RANK signaling in *BRCA1*-breast cancer development, it is necessary to evaluate whether levels of circulating RANKL also predict cancer risk. Thus, the goal of this study was to prospectively evaluate whether plasma RANKL levels are associated with the risk of breast cancer among women with a *BRCA1* or *BRCA2* mutation.

## RESULTS

There was a total of 184 women with a *BRCA1* or *BRCA2* mutation included in the current study. Among all the women combined, the mean plasma RANKL levels were 5.54 pg/ml (range 1.78-14.97). Table 1 summarizes the baseline characteristics of the women by median plasma RANKL (i.e., low RANKL < 5.24 pg/ml and high RANKL ≥ 5.24 pg/ml). The date of blood draw was significantly earlier among women with high vs. low plasma RANKL levels (2004.4 vs. 2006.4; *P* = 0.01). As a result, the length of storage of the blood sample prior to quantification of RANKL was also significantly longer among women with high vs. low RANKL levels (13.60 vs. 11.68 years; *P* = 0.01). All other baseline characteristics were similar between the two groups including reproductive factors, oophorectomy, menopausal status and BMI.

**Table 1:** Characteristics of *BRCA* mutation carriers by median RANKL levels

Variable	Low (n =92)	High (n =92)	*P*^*§*^
Plasma RANKL, pg/ml	3.77 (1.78-5.23)	7.32 (5.25-14.97)	<0.0001
Date of blood draw, mean (range)	2006.4 (1996.0-2012.8)	2004.4 (1997.1-2014.5)	0.01
Sample storage time, mean (range), years	11.68 (5.27-22.03)	13.60 (3.44-20.91)	0.01
Age at blood, mean (range), years	41.3 (19.0-72.2)	43.9 (17.6-86.5)	0.18
Mean follow-up, years (range)	6.2 (0.35-18.8)	6.5 (0.02-19.2)	0.63
*BRCA* mutation type, n (%)			
*BRCA1*	49 (53.3)	60 (65.3)	0.18
*BRCA2*	42 (45.7)	32 (34.8)	
*BRCA1* and *BRCA2* or missing^2^	1 (1.1)	0	
Oophorectomy, n (%)^3^	59 (64.1)	60 (65.2)	0.88
Breast cancer diagnosis, n (%)			
No	85 (92.4)	80 (87.0)	0.23
Yes	7 (7.6)	12 (13.0)	
Age at diagnosis, mean (range)	50.2 (35.5-69.3)	53.4 (33.2-72.4)	0.60
Parity, n (%)			
Nulliparous	30 (32.6)	34 (37.8)	
Parous	62 (67.4)	56 (62.2)	0.47
Mean (range)	1.6 (0-7)	1.6 (0-7)	0.91
Missing	0	2	
Breastfeeding, n (%)			
Never	42 (45.7)	47 (52.2)	
Ever, < 12 months	22 (23.9)	22 (24.4)	0.53
Ever, ≥ 12 months	28 (30.4)	21 (23.3)	
Mean (range), months	9.0 (0-86)	8.6 (0-126)	0.86
Missing	0	1	
Oral contraceptive use, n (%)			
Never	18 (19.6)	24 (26.1)	
Ever	73 (79.4)	68 (73.9)	0.36
Missing	1 (1.1)	0	
Body mass index, kg/m^2^, mean (range)	24.8 (17.9-40.0)	25.3 (18.0-44.2)	0.54
Tamoxifen use, n (%)			
Never	92 (100)	90 (97.8)	
Ever	0	2 (2.2)	0.25
Menopausal status, n (%)			
Premenopausal	66 (71.7)	65 (70.7)	
Postmenopausal	26 (28.3)	27 (29.4)	0.87

^1^*P* values were calculated using the Student's *t*-test for continuous variables and the chi-square test for categorical variables. All tests are two-sided; missing data in not involved in the frequency test.

^2^Women with both a *BRCA1* and *BRCA2* mutation were coded as missing.

3Oophorectomy refers to bilateral oophorectomy.

**Figure 1:**
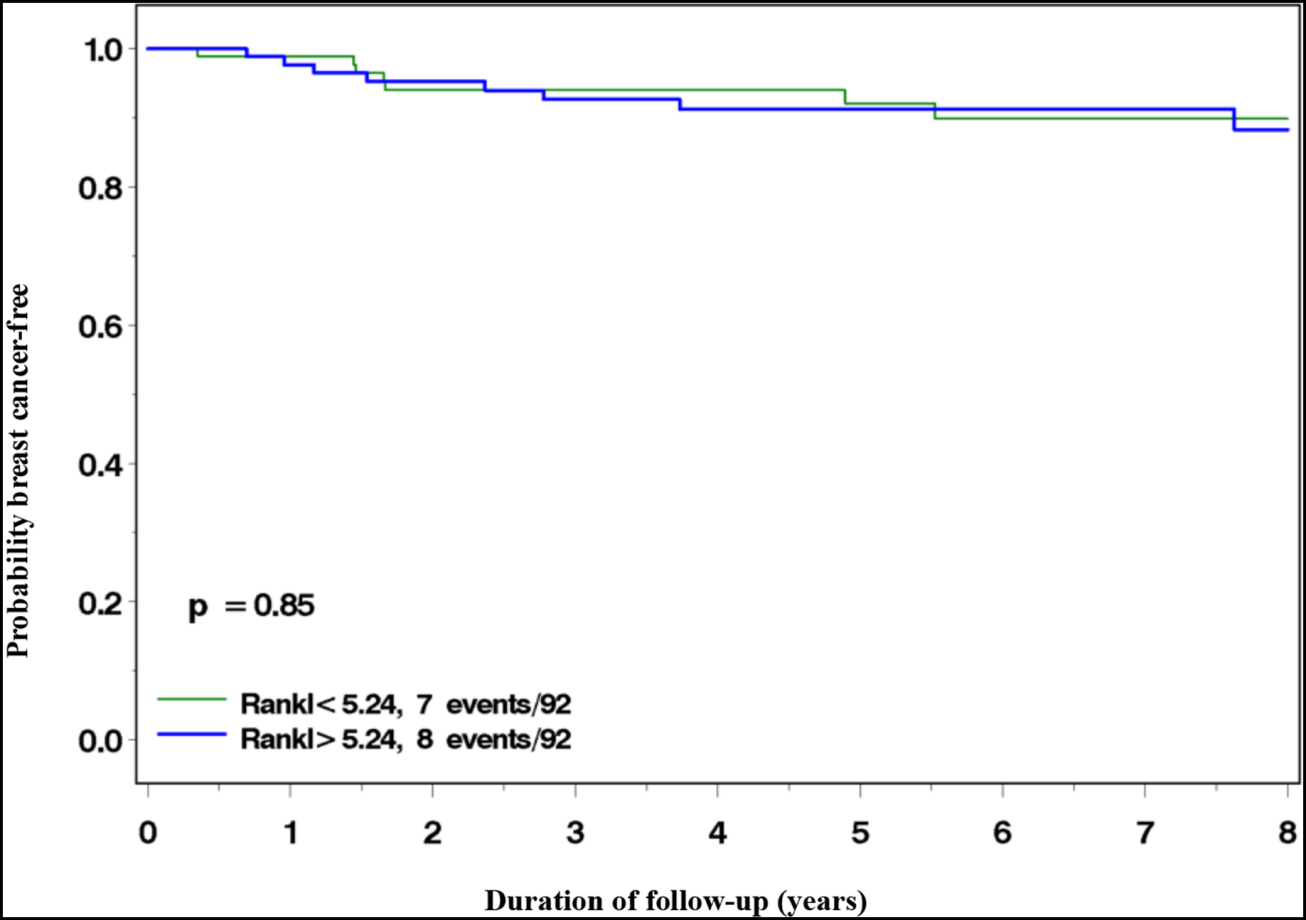
Incidence of breast cancer among *BRCA1* and *BRCA2* mutation carriers with high (>5.24 pg/ml) vs. low (≤5.24 pg/ml) plasma RANKL levels.

There were 15 incident breast cancer cases identified over the mean follow-up period of 6.3 years (0.02-19.24); seven cases (47%) among women in the low RANKL group and 8 (53%) among women in the high RANKL group. The eight-year cumulative incidence was 10% in the low RANKL group and 12% in the high RANKL group (*P* log-rank = 0.85) (Figure 1). Table 2 summarizes the age-adjusted and multivariate hazard ratios (HR) and 95% confidence intervals (CI) of breast cancer associated with high vs. low plasma RANKL levels. In the analysis only adjusted for age at blood draw, the HR was 1.04 (95%CI 0.38-2.89; *P*-trend =0.86). The results did not change substantially in the multivariate model (HR = 1.06; 95%CI 0.34-3.28; *P*-trend =0.86).

**Table 2:** Hazard ratio (HR) and 95% confidence interval (CI) of breast cancer by plasma RANKL levels

Variables	Age-adjusted^1^ HR (95%CI)	*P*	Multivariate^2^ HR (95%CI)	*P*
RANKL, pg/ml				
Low	1.00 (reference)		1.00 (reference)	0.92
High	1.04 (0.38-2.89)	0.93	1.06 (0.34-3.28)	0.86
*P*-trend		0.86		
Age at blood draw, continuous			1.04 (0.98-1.09)	0.18
*BRCA* mutation type				
*BRCA1*			1.00 (reference)	
*BRCA2*			1.61 (0.52-5.04)	0.41
Oophorectomy				
Never			1.00 (reference)	
Ever			0.27 (0.08-0.92)	0.04
Breastfeeding				
Never			1.00 (reference)	
<1 year			0.75 (0.16-3.49)	0.71
≥1 year			0.59 (0.11-3.16)	0.54
Oral contraceptive use				
Never			1.00 (reference)	
Ever			2.19 (0.48-9.98)	0.31
Parity, per birth			1.33 (0.77-2.32)	0.31
Date of blood draw, continuous			1.00 (0.90-1.12)	0.99

^1^Adjusted for age at blood draw.

^2^Adjusted for age at blood draw as well as other variables in the model (*BRCA* mutation, oophorectomy, breastfeeding and oral contraceptive use, parity and date of blood draw).

Both a plasma OPG and RANKL level was available for 176 of the women in the current study. There was no significant correlation between circulating OPG and RANKL levels (ρ = – 0.09; *P* = 0.23.)

## DISCUSSION

In this analysis of plasma RANKL and breast cancer risk among 184 *BRCA* mutation carriers, we found no evidence for an association between circulating RANKL levels and subsequent risk. After an average of 6.3 years of follow-up and 15 incident cancers, the eight-year cumulative incidence was 10% in the low RANKL group and 12% in the high RANKL group. This difference was not statistically significant. Despite the small sample size, the prospective nature of the analysis along with the ability to adjust for potential confounders suggests that RANKL is likely not a circulating biomarker of breast cancer risk among women with an inherited *BRCA1* or *BRCA2* mutation. Furthermore, we found no correlation between circulating levels of RANKL and OPG.

To our knowledge, this represents the first report of plasma RANKL and breast cancer specifically in this high-risk population. In contrast, there have been two recent similar reports conducted among women from the general population [12, 17]. In the first, Kiechl *et al.*, assessed the relationship between tertiles of circulating RANKL, OPG, and progesterone with breast cancer risk among 278 postmenopausal women enrolled in the UK Collaborative Trial of Ovarian Cancer Screening study (UKCTOCS) [17]. In this analysis, 40 women provided blood five to 12 months prior to their breast cancer diagnosis, 58 women provided blood 12 to 24 months prior to their diagnosis and 180 women who did not develop disease served as controls. All breast cancers were estrogen receptor positive (ER+). Among women diagnosed 12 to 24 months following blood collection, high RANKL (as well as high progesterone) was associated with a significantly increased risk of developing breast cancer (odds ratio [OR] = 4.76; 95%CI 1.3-22.8); however, they found no association among women diagnosed within 12 months of blood collection (OR = 1.14; 95%CI 0.3-4.5). The authors concluded that in the setting of high progesterone, high RANKL levels 12-24 months prior to diagnosis (but not 5-12 months prior to diagnosis) was predictive of subsequent risk. This study had several limitations, in particular the stratified analyses resulting in small strata and risk estimates with large confidence intervals.

In a case-control analysis nested within the European Prospective Investigation into Cancer and Nutrition (EPIC) cohort which included 1,976 incident invasive breast cancers and the same number of controls, Sarink *et al.*, evaluated circulating RANKL (and RANKL/OPG) with breast cancer risk [12]. In this study, high serum RANKL was associated with a borderline increased risk of ER+ (quartile 5 vs. quartile 1 relative risk [RR] = 1.28; 95%CI 1.01-1.63; *P*-trend = 0.20) but not ER-negative (ER–) disease (quartile 5 vs. quartile 1 RR = 0.87; 95%CI 0.53-1.44; *P*-trend = 0.21). Results were similar with the RANKL/OPG ratio. The increased risk with both RANKL and RANKL/OPG ratio was limited to women with ER+ disease diagnosed after age 50. In this report, the inter-batch CVs was 21.7%, which is substantially higher than the acceptable value of 15% indicative of laboratory precision, and thus, the results should be interpreted with caution. Given the limited amount of evidence and lack of dose-response, it is currently not clear if RANKL is a biomarker for women without an inherited *BRCA* mutation.

Circulating levels of RANKL and OPG were not correlated in our study. In general, little is known regarding the correlation between circulating OPG and RANKL in healthy women. This is not unexpected considering the wide array of hormones and cytokines that are involved in regulating RANK signaling [18]. A potential explanation for this lack of correlation may be that endogenous RANKL levels remain stable over time while OPG levels increase with age [19]. Furthermore, a significant proportion of RANKL is cell bound [20]. Interestingly, Sarink *et al.*, observed a modest inverse correlation between circulating OPG and RANKL in 458 premenopausal controls (r = -0.40, *p* < 0.01). Importantly, Widschwendter *et al.*, demonstrated that both RANKL and OPG levels were aberrant in *BRCA* mutation carriers implicating both proteins in *BRCA*-breast cancer pathogenesis. However, in their study, changes in RANKL levels within the breast were not observed in circulating RANKL levels. Conversely, decreases in OPG levels within the breast were reflected by subsequent decreases in circulating OPG. This finding suggested that a correlation between circulating OPG and RANKL may not exist. Further investigation is needed regarding potential correlations between OPG and RANKL at the tissue level.

With respect to *BRCA*-associated breast cancer, it is not entirely surprising that we found no association between circulating RANKL and risk. In the initial publication by Widschwendter and colleagues, the authors reported significantly lower circulating levels of both RANKL and OPG among the *BRCA* mutation carriers compared to the non-carrier controls [10]. Interestingly, treatment of cynomolgus macaques with estrogen plus progesterone (but not estrogen alone) resulted in significant downregulation of OPG in both the mammary tissue and serum OPG. Conversely, in response to estrogen plus progesterone, RANKL was upregulated in the mammary tissue however, this upregulation was not reflected in the serum. This suggests that circulating OPG, rather than RANKL, is likely a better proxy of breast tissue activity in the RANK signaling pathway. Furthermore, although both RANKL and OPG are measurable in the blood plasma, RANKL levels are considerably lower than OPG, increasing the potential for samples to be below the limit of detection and given the higher circulating levels, OPG has been suggested to be a better measure of RANK signaling activity than RANKL concentrations [21].

Emerging experimental and epidemiologic data strongly implicate progesterone-mediated RANK signaling in *BRCA1*-breast cancer development [8, 9]. Two groups independently demonstrated that activation of progesterone-mediated RANK signaling in *Brca1* mouse models resulted in the expansion of mammary stem cells (MaSCs) and mammary tumorigenesis [8, 9]. Of particular interest is that RANK is exclusively expressed on luminal progenitor cells within the human breast and that *BRCA1* mutation carriers have an expanded pool of *RANK*^+^ luminal progenitor cells [22]. These cells are hypothesized to be the foundation for the formation of basal-like breast cancers that are frequently observed in women with a *BRCA1* mutation [8, 22]. In addition to an expanded *RANK*^+^ luminal progenitor population, Widschwendter *et al.*, demonstrated that *BRCA* mutation carriers have 121% higher levels of circulating progesterone compared to non-carriers – this may lead to increased activation of progesterone-mediated RANK signaling [23]. Collectively, these findings implicate the progesterone-mediated RANK pathways as a potential target for *BRCA* chemoprevention.

While experimental data strongly support a role of RANKL in breast carcinogenesis, the action of RANKL can be inhibited by OPG, the endogenous decoy receptor for RANKL that antagonizes RANK signaling [2]. In a prospective study of 206 *BRCA* mutation carriers with an average follow-up of 6.5 years, we recently reported a significant inverse relationship between plasma OPG levels and breast cancer risk [11]. Women with high plasma OPG (>median) had a significantly decreased risk of developing breast cancer compared to women with low OPG (<median)(HR=0.25; 95%CI 0.08-0.78). This represents the first prospective investigation of plasma OPG levels and breast cancer risk in women with a *BRCA* mutation and our team is validating these findings in a larger cohort of *BRCA* mutation carriers. Similar to what has been reported among women in the general population, we confirmed a potentially harmful role of progesterone-containing hormone replacement therapy (HRT) [24]. In this first detailed, prospective analysis of HRT use by formulation type after oophorectomy and breast cancer risk in *BRCA1* mutation carriers, we reported cumulative incidence of breast cancer of 22% among women who used estrogen plus progesterone HRT compared to 12% among women who used estrogen-alone HRT (*P*-log rank=0.04).

*BRCA1* mutation carriers face extremely high lifetime risks of breast cancer estimated to be 60% by age 70, although penetrance estimates up to 85% have been reported [25–27]. Prophylactic mastectomy is the most effective risk reduction strategy although uptake is suboptimal [28, 29] and most women opt for intensified screening, which includes yearly MRI and mammography [30]. Despite the identification of this gene over 20 years ago, there are no effective non-surgical strategies. Altogether, the aforementioned studies strongly suggest that inhibition of the RANK signaling pathway with denosumab is a plausible candidate for primary prevention [31]. Indeed, such primary prevention trials are currently being proposed.

Our study was not without limitations. First, was the relatively small sample size which did not allow for stratified analyses by various factors including *BRCA* mutation type; however, the latter was not a significant predictor of RANKL levels in the cox proportional hazards model. We only assessed plasma RANKL at one time point using blood which was collected, on average, 12.64 years (range 3.44-22.03) previously and may not reflect changes in RANKL concentrations over time. In a reproducibility study, which included 221 women enrolled in the EPIC cohort with blood collected at baseline and 14-15 years after recruitment, Fortner *et al.*, reported spearman correlation coefficients of 0.60 and 0.38 for RANKL after one year and 14 years, respectively [32]. Corresponding coefficients for OPG were 0.85 and 0.75, indicative of higher reproducibility over time and suggest that RANKL may not be as reliable a biomarker as OPG and that a single measure of OPG may reflect longer-term levels. Nonetheless, these were not women with an inherited mutation who clearly have inherently aberrant levels of both OPG and RANKL. Although based on self-report, our collection of detailed information on various exposures allowed for adjustment for potential confounders including date of blood collection.

The lack of an association between RANKL and *BRCA* breast cancer risk suggests that circulating RANKL may not be a reliable biomarker of breast cancer risk in this high-risk population, although this requires confirmation in a larger sample of women. Despite this, the current findings reinforce our earlier notion that OPG may serve as a biomarker of breast cancer risk among women with a *BRCA* mutation, identifying those at the highest risk of developing disease. Inherently lower levels of circulating OPG in mutation carriers, which reflects less inhibition of RANK signaling, has been shown in experimental and epidemiologic studies to be important for *BRCA*-associated breast cancer development. The integration of circulating OPG levels into existing risk prediction models may enable the identification of those women who might benefit most from chemoprevention with RANKL blockade.

## MATERIALS AND METHODS

### Study population

This study population has previously been described [11, 16]. Briefly, women enrolled in a longitudinal study of *BRCA1* or *BRCA2* mutation carriers and who had a blood sample available were potentially eligible for inclusion. These women sought testing for *BRCA* mutations because of a personal or family history of breast and/or ovarian cancer. Mutation detection was conducted using a range of techniques, but all nucleotide sequences were confirmed by direct sequencing of DNA. The study was approved by the institutional ethics review boards of the host institutions and all study subjects provided written informed consent. For the current prospective analysis, we only included women who received genetic counselling and testing at Women’s College Hospital (Toronto, Canada).

### Data and sample collection

Each subject completed a baseline questionnaire at the time of a clinic appointment or at their home. The baseline questionnaire collected detailed information on family or personal history of cancer, reproductive and medical histories, as well as information on selected lifestyle factors. Follow-up questionnaires were completed every two years thereafter to update information on relevant exposures and to ascertain incident disease. Blood samples were collected at the time of genetic testing by venipuncture, processed and separated into plasma and DNA and stored at -80°C and 4°C respectively.

### Study subjects available for analysis

Women were eligible for inclusion in the current study if they were between the ages of 18-70 years at the time of enrolment and completed at least one follow-up questionnaire. Of the 743 women who were initially eligible, we excluded those who had a previous history of breast, ovarian or other cancer (n=293), those who had previously undergone a prophylactic bilateral mastectomy (n=16), and those who did not have a baseline plasma sample available (n=250). After these exclusions, a total of 184 participants were available for the current analysis.

### RANKL quantification

Plasma RANKL was quantified using a commercial ELISA kit from ALPCO (Salem, NH – catalogue #04-BI-20462) according to the manufacturer’s protocol. All plasma samples were run in duplicate. The RANKL concentration (pg/ml) was calculated as the average of duplicate samples (each adjusted for background signal and normalized to blank wells) and subsequently converted to a total RANKL concentration upon comparison to RANKL standards provided in the kit. A common quality control (QC) sample was included on every plate. The average intra-assay coefficient of variation (CV) was approximately 2.7%. This was calculated using the mean CV of duplicate samples within each plate. The average inter-assay CV was 3.4%. This was calculated using the common QC sample run on each plate.

### Statistical analysis

Women were categorized into high or low plasma RANKL based on the median levels in the entire cohort (≤5.24 and >5.24 pg/ml). Baseline characteristics of the women with high vs. low RANKL levels were compared using the student’s *t*-test and Ӽ^2^ test. Participants were followed from date of the baseline questionnaire until either date of: 1) breast cancer, 2) prophylactic mastectomy, 2) ovarian cancer, 3) death or 4) completion of last follow-up questionnaire. The follow-up period of this analysis was from the date of baseline questionnaire until November 25^th^, 2017. Kaplan-Meier survival analysis was used to estimate the eight-year cumulative incidence of breast cancer in women with high vs. low RANKL levels and compared using a log-rank test. Cox proportional hazards models to estimate the hazard ratio (HR) and 95% confidence interval (CI) associated with plasma RANKL levels adjusted for age at blood draw (continuous). The multivariate analysis was further adjusted for date of blood draw (continuous), *BRCA* mutation type (*BRCA1* or *BRCA2*), oophorectomy (yes, no), breastfeeding (never, <1 year, ≥ 1 year), oral contraceptive use (never, ever), and parity (never, 1, 2, 3, ≥4). All analyses were conducted using SAS version 9.4 software (SAS Institute, Cary, NC, USA). All *P* values were 2-sided and were considered statistically significant if *P* ≤0.05.
